# Fabrication of Functional Polymers with Gradual Release of a Bioactive Precursor for Agricultural Applications

**DOI:** 10.3390/gels11020090

**Published:** 2025-01-24

**Authors:** Oscar G. Marambio, Rudy Martin-Trasancos, Julio Sánchez, Felipe A. Ramos, Guadalupe del C. Pizarro

**Affiliations:** 1 Departamento de Química, Facultad de Ciencias Naturales, Matemáticas y Medio Ambiente, Universidad Tecnológica Metropolitana (UTEM), J. P. Alessandri 1242, Santiago 7800002, Chile; framos@utem.cl; 2 Departamento de Química de los Materiales, Facultad de Química y Biología, Universidad de Santiago de Chile (USACH), Santiago 9170002, Chile; rudy.martin@usach.cl; 3 Departamento de Química Orgánica, Facultad de Química y de Farmacia, Pontificia Universidad Católica de Chile, Santiago 7820436, Chile; julio.sanchez@uc.cl

**Keywords:** controlled herbicide release system, ultrafiltration system, polymeric synthetic hydrogel

## Abstract

Biodegradable and biocompatible polymeric materials and stimulus-responsive hydrogels are widely used in the pharmaceutical, agricultural, biomedical, and consumer sectors. The effectiveness of these formulations depends significantly on the appropriate selection of polymer support. Through chemical or enzymatic hydrolysis, these materials can gradually release bioactive agents, enabling controlled drug release. The objective of this work is to synthesize, characterize, and apply two controlled-release polymeric systems, focusing on the release of a phyto-pharmaceutical agent (herbicide) at varying pH levels. The copolymers were synthesized via free radical polymerization in solution, utilizing tetrahydrofuran (THF) as the organic solvent and benzoyl peroxide (BPO) as the initiator, without the use of a cross-linking agent. Initially, the herbicide was grafted onto the polymeric chains, and its release was subsequently tested across different pH environments in a heterogeneous phase using an ultrafiltration (UF) system. The development of these two controlled-release polymer systems aimed to measure the herbicide’s release across different pH levels. The goal is to adapt these materials for agricultural use, enhancing soil quality and promoting efficient water usage in farming practices. The results indicate that the release of the herbicide from the conjugate systems exceeded 90% of the bioactive compound after 8 days at pH 10 for both systems. Furthermore, the two polymeric systems demonstrated first-order kinetics for herbicide release in aqueous solutions at different pH levels. The kinetic constant was found to be higher at pH 7 and 10 compared to pH 3. These synthetic hydrogels are recognized as functional polymers suitable for the sustained release of herbicides in agricultural applications.

## 1. Introduction

Modern agriculture is under growing pressure to improve crop productivity while reducing its environmental impact. Although modern agriculture offers numerous solutions, the results can vary significantly because each farm is unique, with different landscapes, soil types, available technologies, and potential yields [1]. The past few decades have seen significant growth driven by plant protection products and other technological innovations. However, the excessive use of these inputs has a negative impact on the environment [2]. Traditional approaches to herbicide use can be beneficial when applied carefully. However, inappropriate or excessive application can lead to various issues, such as chemical residues, phytotoxicity in crops, and negative impacts on subsequent or susceptible plants and non-target organisms. Additionally, the repeated use of similar types of herbicides over the years can lead to accumulation in crops, soil, and groundwater, potentially posing health risks [3].

Weeds are a major factor in reducing the yields of many crops, and herbicides are the most commonly used pesticides [4]. The herbicide 2,4-D is a phenoxy herbicide that functions as a synthetic plant hormone. It effectively and selectively targets broadleaf weeds in cereal crops while leaving legumes and corn unharmed. Additionally, 2,4-D is compatible with most pesticides commonly used in agriculture [5].

Recently, increasing interest has been in designing and fabricating “smart” hydrogels that respond to external stimuli such as pH, pressure, light, temperature, ionic strength, and enzymes [6]. This interest stems from hydrogels’ excellent biocompatibility, straightforward preparation, and a broad range of applications [7]. For example, they have been used as functional materials in drug delivery [8,9] and tissue engineering [10]. Polymers that support agricultural chemicals have been developed to address the significant environmental issues associated with conventional agrochemicals [9,11,12,13,14,15,16,17,18,19,20,21,22,23].

The delivery of herbicides through controlled-release formulations provides both ecological and economic benefits [13]. The effectiveness of these formulations relies on selecting an appropriate polymer support. Degradable polymer materials and hydrogels have various applications in pharmaceutical, agricultural, biomedical, and consumer-oriented fields, making them significant for controlled-release systems [24,25,26,27,28].

Conversely, numerous studies have examined how organic polymers affect soil physical properties [29]. Soil conservation has improved through the addition of polymeric materials to irrigation systems [30,31,32]. Hydrophilic polymers, including poly(vinyl alcohol) (PVA), carboxymethylcellulose, poly(acrylamide) (PAM), and hydrolyzed starch-g-poly(acrylonitrile) copolymers (HSPAN). These polymeric materials have been suggested as soil conditioners [33,34]. The successful use of polyacrylamide (PAM) in irrigation water has generated interest in exploring other polymers with similar properties, especially since acrylamide, the monomer used to create PAM, is known to be a neurotoxin. Although using PAM that contains less than 0.05% monomer helps alleviate some concerns, it does not fully address the potential risk of monomer formation as a degradation product, particularly because the amine group can be removed from the polymer backbone during degradation [35]. Interest in agricultural polymers’ final fate and their degradation products’ potential ecotoxicity is increasing [36,37,38]. These materials have interconnected polymer networks that respond to specific stimuli [39,40,41,42]. Furthermore, it has been observed that hydrogels’ hydration and adsorption capacity is influenced by factors such as the medium’s pH, ionic strength, and osmotic pressure.

Our research group has shown that acrylate-based hydrogels excel in this type of application due to their high reactivity, obtained through free radical polymerization [43,44,45]. Drug release from hydrogel networks is influenced by various mechanisms, such as matrix swelling, drug dissolution and diffusion, and hydrogel erosion. The two main chemical controlled-release systems are erodible drug delivery systems [46,47] and pendant chain systems [48,49]. The drug is connected to the polymer backbone via degradable linkages in pendant chain systems. As these linkages break down, the drug is released gradually. The drug can be attached either directly to the polymer or through a “spacer” group. It is uniformly dispersed throughout the polymer, and the drug is released slowly as the polymer disintegrates [49]. The degradation rate of polymer–drug linkages affects drug release. Typically, these linkages undergo hydrolysis, allowing the degradation and release rates to be described using first-order kinetic relationships [48]. In certain applications, the drug–polymer linkages may be designed to be degradable by enzymes, resulting in more complex release kinetics [27,49].

In this report, we present two chemical controlled-release systems based on synthetic poly(2-hydroxyethylmethacrylate-*alt*-maleic anhydride)[P(HEMA-*alt*-MAn)] and poly(2-hydroxypropylmethacrylate-*alt*-maleic anhydride)[P(HPMA-*alt*-MAn)] as physical hydrogels, in which the functional groups in the polymer support and hydrogels’ unique network structure allow for high levels of hydrophilicity. This work presents several key contributions. First, we designed two controlled-release polymers for a herbicide as a pharmaceutical agent to assess its release in various pH environments. The goal is to adapt these materials for agricultural use, improving soil quality and ensuring efficient water usage, particularly during drought conditions.

Second, the polymers were synthesized using free radical polymerization in solution. An organic solvent was employed, with benzoyl peroxide (BPO) acting as the initiator. The functional groups of the hydrophilic monomers were activated in a basic environment to enhance physical bonds through hydrogen bonding between the chains, leading to the formation of a physical hydrogel. Subsequently, we characterized its structure and incorporated the herbicide into the main chain through an esterification reaction. Finally, we utilized an ultrafiltration system to investigate the controlled release of the herbicide from the prepared system. We studied the release characteristics of these systems in neutral, alkaline, and acidic media.

These degradable polymeric hydrogels hold potential for a wide range of agricultural applications, making them particularly significant for controlled-release systems. The copolymer hydrogels and copolymer–herbicide conjugates were analyzed using FTIR, ^1^H NMR spectroscopy, and TG analyses. Additionally, their excellent soft physical properties linked to the degradation process make them ideal agricultural materials.

## 2. Results and Discussion

### 2.1. Characterization of the Alternating Copolymers

**Figure 1:**
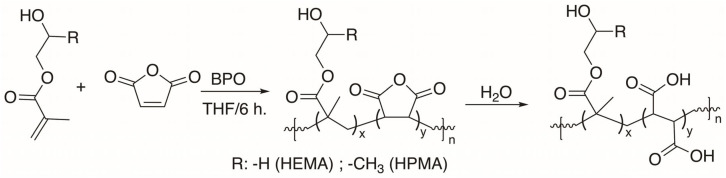
Polymerization reaction of the P(HEMA-*alt*-MAn) and P(HPMA-*alt*-MAn) and copolymers under hydrolysis conditions.

It is widely recognized that interesting alternating structures can be formed through the copolymerization of maleic anhydride (MAn) with vinyl monomers. The effectiveness of this process depends on the reactivity of the double bonds in the vinyl monomers and the specific reaction conditions used. When MAn is copolymerized with vinyl monomers that have electron-donating characteristics using conventional radical techniques, it can create alternating copolymers. The copolymers were synthesized through free radical polymerization, following the conditions outlined in Table 1. These copolymers resulted in alternating structures, P(HEMA-*alt*-MAn) and P(HPMA-*alt*-MAn), as depicted in Figure 1. The molecular weight (Mw) of Poly(HEMA-*alt*-MAn) was 14,200, while that of Poly (HPMA-*alt*-MAn) was 16,580. With a polydispersity (Ð = Mw/Mn) of 1.45 and 1.53, respectively, the molecular weight (Mw) ratios were determined using GPC.

The hydrolysis of the anhydride group leads to carboxylic acid, which is responsible for the hydrogel properties and swelling behavior.

**Figure 2:**
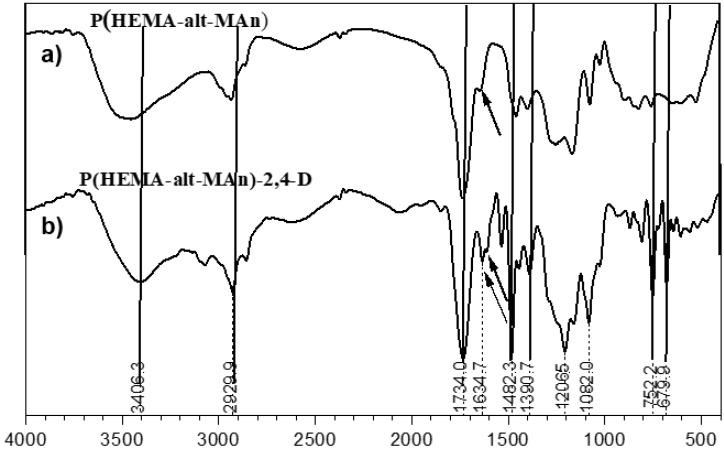
(**a**) FTIR spectrum of P(HEMA-*alt*-MAn) and (**b**) its copolymer conjugate 2,4-D.

**Figure 3:**
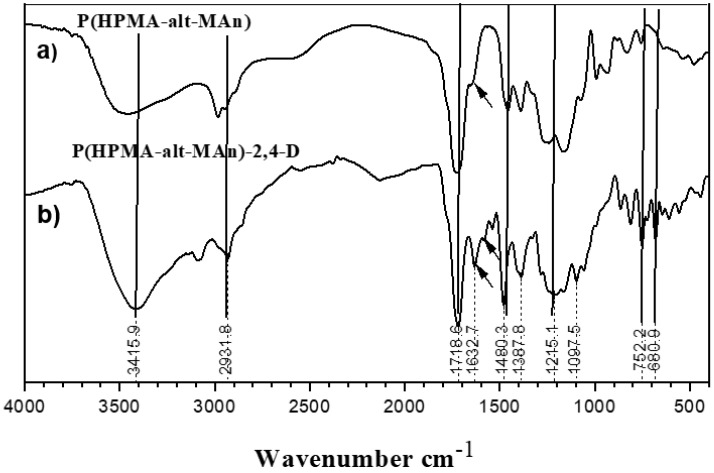
(**a**) FTIR spectrum of P(HPMA-*alt*-MAn) and (**b**) its copolymer conjugate 2,4-D.

The characterization of the P(HEMA-*alt*-MAn) by FTIR (KBr, cm^−1^) exhibited the following signals: 3449.7 ν(OH); 2930.9 ν(CH, CH_2_); 1734.0 ν(C=O, for maleic anhydride, MAn); 1634.7.ν(C=O, for HEMA); and 1482.3 and 1390.7 ν(CH_2_-; CH_3_-); see Figure 2a. FTIR (KBr, cm^−1^) of P(HPMA-*alt*-MAn) exhibited the following signals: 3415.9. ν(OH); 2931.8 ν(CH, CH_2_); 1718.6 ν(C=O, ester for MAn); 1632.7 ν(C=O, ester for HPMA); and 1480.3 and 1387.8 ν(CH_2_-; CH_3_-); see Figure 3a.

### 2.2. Characterization of the Copolymers Grafted with the Herbicide

**Figure 4:**
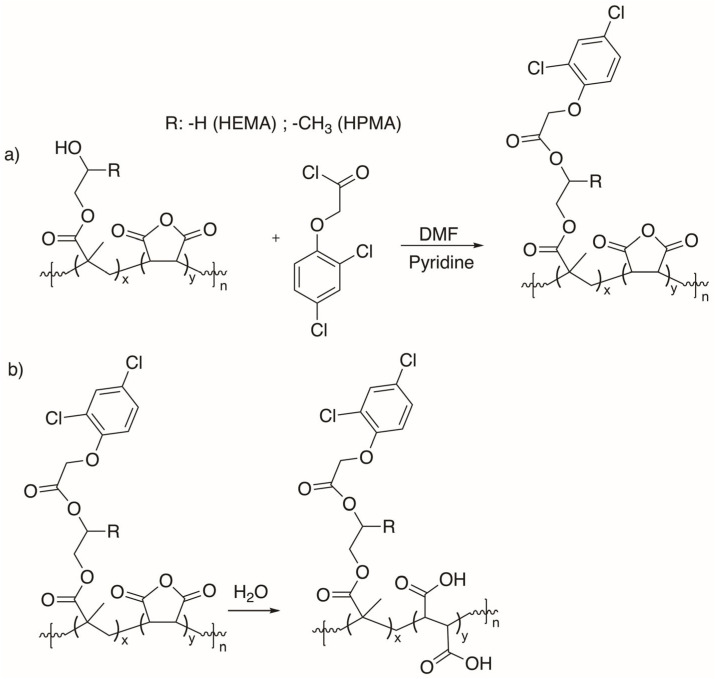
(**a**) Grafting reaction of 2,4-D onto P(HEMA-*alt*-MAn) and P(HPMA-*alt*-MAn) and (**b**) the corresponding hydrolysis of the anhydride upon swelling.

The grafting procedure using the herbicide 2,4-dichlorophenoxyacetic chloride was carried out according to the reaction shown in Figure 4 and the experimental conditions in Table 2.

FTIR (KBr, cm^−1^) of P(HEMA-*alt*-MAn)-2,4-D exhibited the following signals: 3406.3 ν(OH); 2929.9 ν(CH, CH_2_); 1734.0, 1634.7, and 1616.3 assigned at ν(C=O, ester from MAn, HEMA-2,4-D, respectively), and an increase in signal intensity was observed, along with a slight shift towards a shorter wavenumber; and 1482.3 and 1390.7 ν(CH_2_-; CH_3_-); and 752.2 and 679.9 ν(C=C, aromatic ring) from herbicide; see Figure 3b. FTIR (KBr, cm^−1^) of P(HPMA-*alt*-MAn)-2,4-D: 3415.9 ν(OH); 2931.8 ν(CH, CH_2_); 1781.1, 1632.8, and and 1622.6 assigned at ν(C=O, ester from MAn, HPMA-2,4-D, respectively), and an increase in signal intensity was observed, along with a slight shift towards a shorter wavenumber; 1480.3 and 1387.8 ν(CH_2_-; CH_3_-); 752.2 and 680.9 ν(C=C, aromatic ring) from herbicide; see Figure 4b [9,50,51].

**Figure 5:**
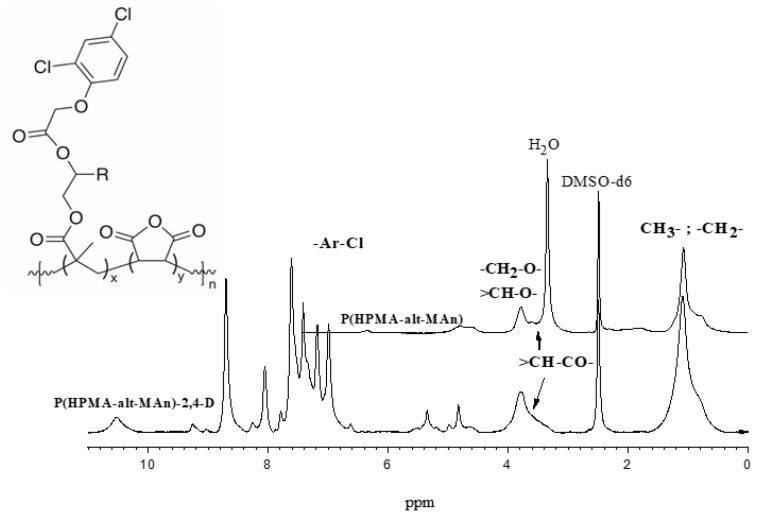
^1^H-NMR spectrum of P(HPMA-*alt*-MAn) and its copolymer conjugate 2,4-D.

^1^H-NMR (δ in ppm) showed the following signals: 0.3–2.2, [-CH_3_, -CH_2_ from backbone of HEMA], 2.6–5.4 [-CH-CO- of MAn], [-CH_2_-O- from lateral chain of HEMA], and 6.4 [-OH lateral chain]. On the other hand, the characterization of the P(HPMA-*alt*-MAn) by ^1^H-NMR (δ in ppm) showed the following signals: 0.6–1.6, [-CH_3_, -CH_2_ from backbone, and -CH_3_, of lateral chain of HPMA] and 3.4–4.2 [−CH_2_-O, and -CH-O- from HPMA and -CH-CO- from MA]; see Figure 5. The ^1^H-NMR spectrum (δ in ppm) of P(HPMA-*alt*-MAn)-2,4-D exhibited the following signals: 0.6–1.2, [-CH_3_, -CH_2_ from backbone of HEMA], 3.6 [-CH-CO- of MAn], 4.16 [-CH_2_-O- from lateral chain of HPMA], 4.83–4.93 [-CH_2_O-Ar from 2,4-D unit], and 6.8–7.7 [-CH aromatic ring from the herbicide, 2,4-D]; see Figure 5 [9,50,51].

The ^1^H-NMR analyses also showed increasing concentration or intensity of the C=O band of the esterification, since the signal at 4.83–4.93 is assigned to -CH_2_O-Ar from the 2,4-D unit and 7.0–7.6 is assigned to the -CH aromatic ring from the herbicide; see Figure 5. Conversely, the FTIR analyses of the copolymers showed typical bands corresponding to carbonyl groups (see Figure 2 and Figure 3). Once the esterification reaction was achieved, the FTIR analyses showed an increase in the intensity of the C=O in the esterification reaction, since the band corresponding to carbonyl groups appears at around 1634.7 cm^−1^, and bending bands corresponding to the aromatic group appear at 752.2 and 679.9 cm^−1^.

### 2.3. Studies of the Thermal Behavior of the Hydrogels

**Figure 6:**
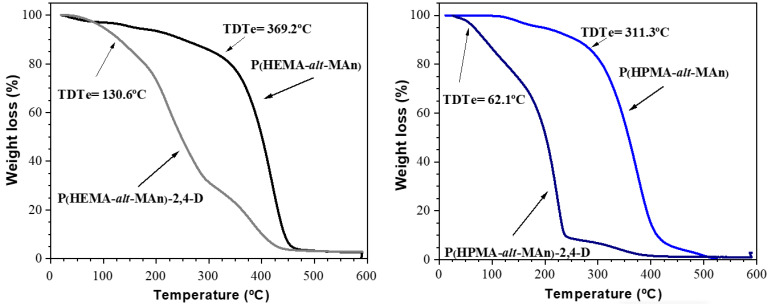
Thermograms of P(HEMA-*alt*-MAn) and its conjugate P(HEMA-*alt*-MAn)-2,4-D (**left**), and P(HPMA-*alt*-MAn) and its conjugate P(HPMA-*alt*-MAn)-2,4-D (**right**). Heating rate, 10 °C min^−1^, in inert environment (gaseous N_2_).

The thermal behavior of P(HEMA-*alt*-MAn) and P(HPMA-*alt*-MAn), along with their conjugates with 2,4-D, was examined using thermogravimetric analysis (TGA). As shown in Figure 6, both the copolymers and their conjugates exhibited consistent degradation patterns, occurring in one-stage and two-stage processes, respectively. The copolymers remained stable up to approximately 300 °C, with a weight loss (WL) of less than 20%. In contrast, the herbicide conjugates exhibited lower thermal stability than their corresponding copolymers, indicating that the covalent bond formed by the ester functional group between the polymer and the herbicide is relatively weak. When comparing the thermal stability of both copolymers, P(HEMA-*alt*-MAn) demonstrated a higher thermal decomposition temperature (TDT) of 369.2 °C, with an average weight loss of 6.2%. In contrast, the TDT for P(HPMA-*alt*-MAn) copolymers was 311.3 °C, accompanied by a weight loss of 19.13%. Furthermore, when examining the thermal stability of the copolymer–herbicide conjugates, the P(HPMA-*alt*-MAn)–herbicide combination exhibited a lower TDT of 62.1 °C (average weight loss of 10.03%) compared to the P(HEMA-*alt*-MAn)–herbicide, which had a TDT, of 130.6 °C (average weight loss of 14.5%). This indicates that the P(HEMA-*alt*-MAn)–herbicide conjugate has higher thermal stability.

### 2.4. Effect of the pH on Swelling Studies

**Figure 7:**
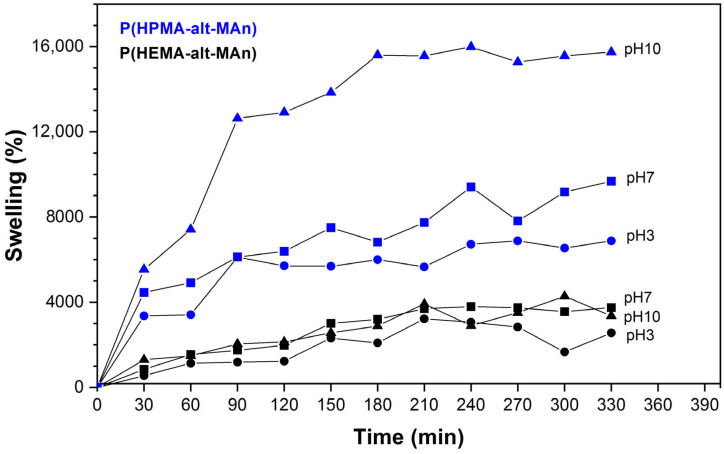
Swelling isotherm of P(HEMA-*alt*-MAn) and P(HPMA-*alt*-MAn) as a function of time in buffered solutions at specific pH levels: 3 (●), 7 (■), and 10 (▲) at 25 °C.

The swelling behavior of P(HEMA-*alt*-MAn) and P(HPMA-*alt*-MAn) copolymers were studied based on their behavior at different pH levels by immersing the gels in buffered solutions at different pH values (3, 7, and 10), all at 25 °C. The results, displayed in Figure 7, show the swelling times corresponding to each condition.

The swelling capacity of P(HPMA-*alt*-MAn) was higher than that of P(HEMA-*alt*-MAn). This can be attributed to the fact that in the HPMA residue, there is a greater balance of hydrophilic and hydrophobic residues, which favors the swelling properties, where the hydroxyl group interacts with the acidic carboxylic group of MAn (acid medium) or carboxylate (basic medium), causing a larger cell size and greater flexibility, which increases the hydration capacity. On the other hand, similar behavior was found for P(HEMA-*alt*-MAn) at pH 3, 7, and 10, while for the P(HPMA-*alt*-MAn) hydrogel, the swelling capacity increased when increasing the pH from 3 to 10. The swelling properties of these polymers depend significantly on the balance between hydrophilic and hydrophobic components. In the case of P(HPMA-*alt*-MAn), the observed pH dependency can be explained by an increase in the hydrolysis of the MAn residues, which generates charged carboxylate groups at higher (basic) pH levels. Conversely, the swelling properties of P(HEMA-*alt*-MAn) do not depend on pH due to a lower hydrophobic–hydrophilic balance of the HEMA residue. Additionally, after the hydrolysis of the MAn residues, the carboxylic groups may stabilize through hydrogen bonding with the hydroxyl groups of HEMA. It is also suggested that this stabilizing effect may be reduced by steric hindrance in the case of HPMA.

### 2.5. Release of 2,4-D Through Heterogeneous Hydrolysis

An ultrafiltration system was employed to analyze the release 2,4-D from copolymer–herbicide conjugates in buffer solutions with pH levels of 3, 7, and 10. To effectively analyze the resulting data, several assumptions need to be established:

(a) The kinetics of hydrolysis of the conjugates is slower than the time taken to collect the filtration fractions, which occurs daily over a period of 3 to 6 min. Therefore, the amount of herbicide released during this short duration is considered negligible.
(b) The herbicide is released from the conjugates into the solution filtrate freely from the ultrafiltration cell. In this case, the concentration of herbicide in each filtration fraction can be related to the concentration of the herbicide free in solution every day (*c^free^*^-*day*^). In the washing method of the LPR technique, as illustrated in Figure 6, when a low-molecular-weight species is filtered out of the ultrafiltration system and no interaction occurs between this species and the components of the ultrafiltration cell, including the ultrafiltration membrane, the instantaneous concentration of this species in the filtrate is determined by the following Equations (1)–(9), previously reported [9]. These represent the mass balance in releasing the 2,4-D herbicide in ultrafiltration.

(1) $$c^{filtrate}=c^{filtrate-init}\exp(-F)$$

When *F* is defined as the filtration factor *F* = *V^filtrate^*/*V^cell^*, it refers to the measure of how well a substance or fluid can pass through a filter or porous material.

The concentration of 2,4-D in the filtration fractions (*c^filtrate^*) is a mean value considering the instantaneous concentrations in the filtration process (*c^filtrate^*), which decrease during filtration. Making a mass balance, we have that

(2) $$\int_{F=0}^{F}c^{filtrate}dF=<c^{filtrate}>\Delta F$$

substituting (1) in (2) we obtain

(3) $$\int_{F=0}^{F}c^{filtrate-init}\exp(-F)dF=<c^{filtrate}>\Delta F$$

and integrating both equations

(4) $$c^{filtrate-init}=\frac{<c^{filtrate}>\Delta F}{1-e^{-F}}$$

As the filtration fraction obtained every day consists of 20 mL, so that final *F* = 1 and Δ*F* = 1, it is found that

(5) $$c^{free-day}=c^{filtrate-init}=\frac{<c^{filtrate}>}{1-e^{-1}}$$

Thus, *^free^*^-*day*^ corresponds to the concentration of the first differential volume obtained in the collection process (*c^filtrate^*^-*init*^), and is calculated from the value of <*c^filtrate^*> corrected by Equation (5).

**Figure 8:**
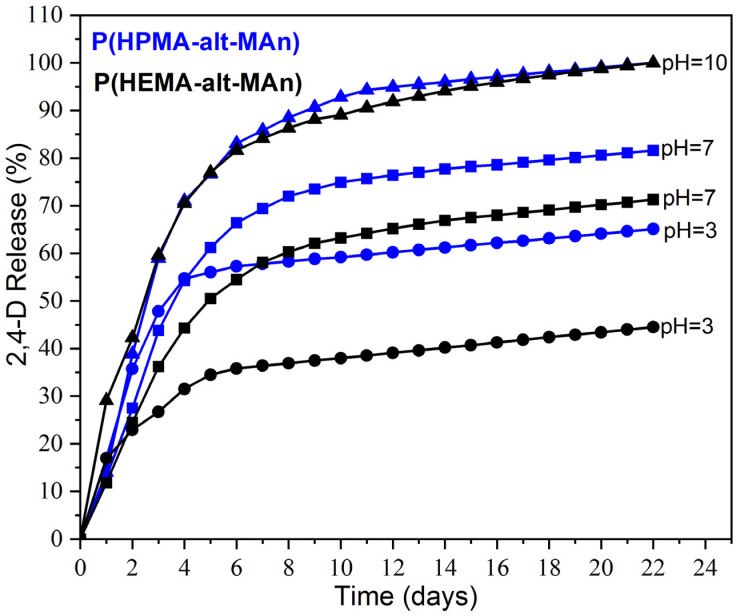
Release of 2,4-D (%) from copolymer–herbicide conjugates in an aqueous solution at pH 3, 7, and 10.

The concentration of herbicide bound to the polymer every day (*c^bound^*^-*day*^) is given by

(6) $$c^{bound-day}=c^{cell-day}-c^{free-day}=c^{cell-day}-\frac{<c^{filtrate}>}{1-e^{-1}}$$

where *c^cell^*^-*day*^ is the concentration of herbicide in the cell every day and is calculated following

(7) $$c^{cell-day}=c^{cell-init}-\sum_{day}(<c^{filtrate}>\Delta F)$$

where *c^cell^*^-*init*^ is the initial concentration of herbicide in the cell. If the kinetics of the hydrolysis of the herbicide from the conjugate systems is of order one, then there should be found an exponential decay of *c^bound^*^-*day*^ with time, since

(8) $$-\frac{dc^{bound-day}}{dt}=kc^{bound-day}$$

and then

(9) $$c^{bound-day}=c^{bound-day-init}\exp(-kt)$$

where *k* is the release constant. Release profiles of P(HEMA-*alt*-MAn)-2,4-D and P(HPMA-*alt*-MAn)-2,4-D are exhibited in Figure 8. The release rates of both conjugates were notably higher at pH 10, exceeding 85% over a duration of 7 days.

The release of drugs linked by covalent bonds depends on the degradation rate of the polymer–drug connection. Typically, these connections degrade through hydrolysis, allowing the rates of degradation and release to follow simple first-order kinetic relationships [48]. In specific applications, the drug–polymer linkages can be designed to degrade enzymatically, leading to more complex release kinetics [49,52,53].

**Figure 9:**
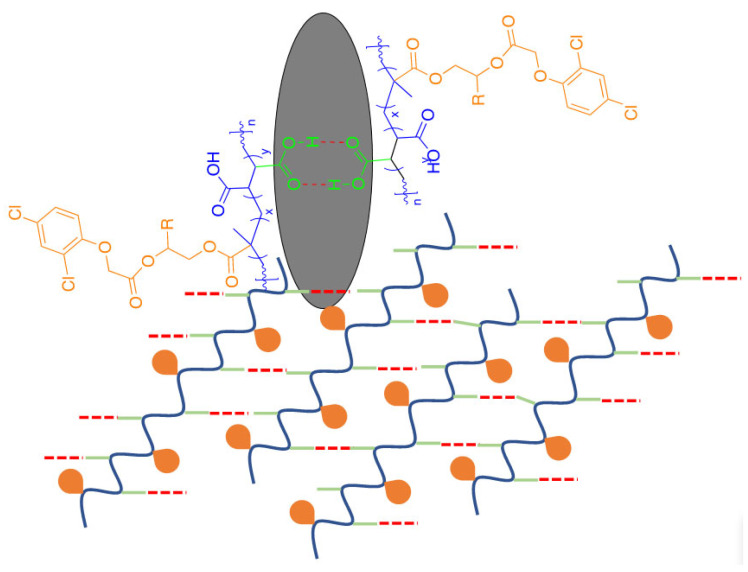
Schematic representation of inter- or intra-hydrogen bonds in fabricating stimulus-responsive hydrogels.

First-order kinetics occur when a constant proportion of the phytopharmaceutical is eliminated per unit of time. The elimination rate is proportional to the amount of phytopharmaceutical in the copolymeric systems, but is pH-dependent; at basic pH, it is increasing. The higher the concentration, the greater the amount of drug eliminated per unit of time at higher pH. For each half-life that passes, the concentration of the drug is halved. The elimination rate decreases proportionally as the drug concentration decreases while maintaining a constant elimination rate, k. The reaction rates are influenced by the olefinic comonomer used, whether it is HEMA or HPMA, to which 2,4-D is grafted. In this way, the positive release of the drug is affected by the increased hydrophilicity and conformational flexibility of the polymer, with the HPMA comonomer. This enhancement occurs due to the extension of the lateral chain of HPMA and the steric hindrance caused by the methyl group, which prevents the formation of inter- or intra-dipole–dipole interactions (hydrogen bonds) between the highly electronegative oxygen (O) atoms of the O–H groups, which is different for the HEMA comonomer; see Figure 9. Additionally, for the HPMA monomer, an increase in the content of hydrophilic co-units, as seen in copolymers containing MAc, contributes to this effect. This suggests that the release of herbicides significantly increases with higher pH levels, primarily because hydrolysis becomes more effective at elevated pH. The notable increase in release at pH 10 indicates that the basic hydrolysis of acetate groups may impact the breakdown of certain herbicides. Lower release values were observed at pH levels of 7 and 3, which aligns with the mechanism of hydrolytic cleavage of the herbicide–polymer bond. The differences in release rates between the two copolymer conjugates at pH 3 and 7 can be explained by their varying swelling capacities, which differ between the HEMA and HPMA comonomers. The release rate typically increases with higher swelling from aqueous solutions, associated with greater polymer hydrophilicity for the P(HPMA-*alt*-MAn) system. The MAn copolymer reacts easily with water to form MAn-MAc terpolymers at basic pH (the maleic anhydride ring is open). With a sufficient amount of water, MAn hydrolyzes completely to MAc copolymers at pH 10. The reaction rates also depend on the olefinic comonomer. Additionally, an increase in the content of hydrophilic co-units, as seen in copolymers containing MAc, contributes to this effect.

Conversely, the positive release is influenced by the increased hydrophilicity and conformational flexibility of the polymer.

**Figure 10:**
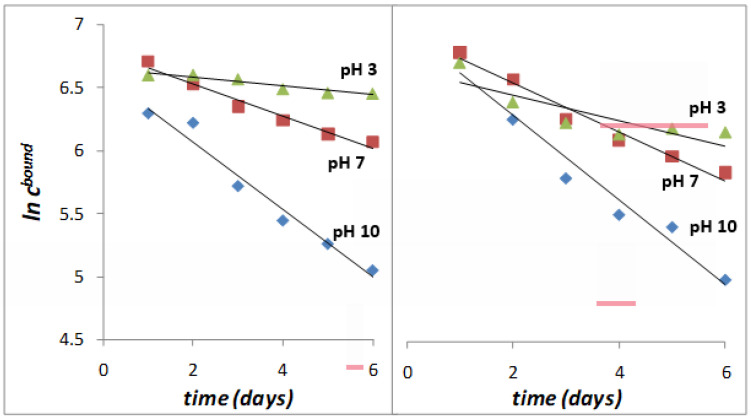
Determination of kinetic release constants at different pH. Plot of ln *c^bound^* vs. time (days).

The kinetic constants for the various systems at different pH values were obtained from the slopes of the plots shown in Figure 10, as indicated by Equation (9). These values are presented in Table 1. The exponential decay observed in the release profiles confirms that the hydrolysis of the copolymer conjugates follows first-order kinetics. It can be seen in Figure 8 the evolution of ln *c ^bound^*^-*day*^ with time during the first 6 days.

The values of R2 for n = 1 for the release of herbicide for P(HPMA-*alt*-MAn) y P(HEMA-*alt*-MAn) at pH 3.0, 7.0, and 10 are shown in the Table 3. These results show that the value of n = 1 is maintained with an increase in pH.

## 3. Conclusions

A series of two pH-sensitive copolymers, P(HEMA-*alt*-MAn) and P(HPMA-*alt*-MAn), have been designed as controlled-release systems using the herbicide 2,4-D. These copolymers demonstrate pH sensitivity, exhibiting a higher swelling ratio in aqueous solutions at elevated pH levels. The release values measured at pH 10 indicate that the cleavage of the herbicide may be influenced by the basic hydrolysis of the acetate groups. In contrast, lower release values were observed at pH 7 and pH 3, consistent with the mechanism of hydrolytic cleavage of the herbicide–polymer bond. The release rate typically increases with a greater swelling from the water solution, which corresponds to enhanced hydrophilicity of the polymer. This phenomenon can be attributed to the increased hydrolysis of the MAn residues and the dissociation of the acidic groups. The release is positively influenced by the increased hydrophilicity of the lateral chain in HPMA (due to the presence of the methyl group). This enhancement occurs because the steric hindrance created by the methyl group prevents the formation of inter- or intra-molecular hydrogen bonds. Additionally, there is an increase in the content of the hydrophilic co-unit (MAc), which results from the esterification of the MAn-containing copolymer. In contrast, the swelling capacity of P(HPMA-*alt*-MAn) is greater than that of P(HEMA-*alt*-MAn). This difference may be due to the higher steric hindrance caused by the methyl group in HPMA. The copolymer functionalized with 2,4-D displayed labile ester bonds that can be cleaved by heating, as demonstrated by TGA analyses. The hydrolysis of the herbicide from the conjugates follows first-order kinetics, with higher kinetic constants observed at increasing pH levels. This indicates that the hydrolysis reaction is base-catalyzed. These findings suggest that pH-sensitive hydrogels could be used as intelligent drug delivery carriers in agriculture.

## 4. Materials and Methods

### 4.1. Materials

2-hydroxyethylmethacrylate (HEMA) and 2-hydroxypropylmethacrylate (HPMA) (Merck, Darmstadt, Germany) were purified by distillation. Benzoyl peroxide (BPO) (Sigma Aldrich, St. Louis, MO, USA) was used as an initiator of the copolymerization reaction between HEMA or HPMA with maleic anhydride (MAn) (Sigma Aldrich, St. Louis, MO, USA). All the other reagents, including the herbicide 2,4-dichlorophenoxy acetic acid (2,4-D) (Sigma Aldrich, St. Louis, MO, USA), were used as received. Thionyl chloride (SOCl_2_) (Sigma Aldrich, St. Louis, MO, USA) was used as received.

### 4.2. Equipment

The structural characterization of the polymers was carried out by proton nuclear magnetic resonance (^1^H-NMR) on a Bruker 200 MHz spectrometer, Karlsruhe, Germany. in DMSO-d_6_, at room temperature. Fourier transform infrared spectroscopy (FTIR) spectra were acquired by a Bruker Vector 22 (Bruker Optics GmbH, Karlsruhe, Germany), in the range of 400 to 4000 cm^−1^. Gel permeation chromatography (GPC) was conducted to determine the number-averaged molecular weight (Mn) and weight-averaged molecular weight (Mw) under the following conditions: a WATERS 600E instrument (Kioto, Japan) equipped with UV and RI detectors, using tetrahydrofuran (THF) as the solvent at a flow rate of 1.0 mL/min. The samples were analyzed at a concentration of 4 mg/mL and at a temperature of 25 °C. Calibration was performed using poly(methyl methacrylate) (PMMA).

**Figure 11:**
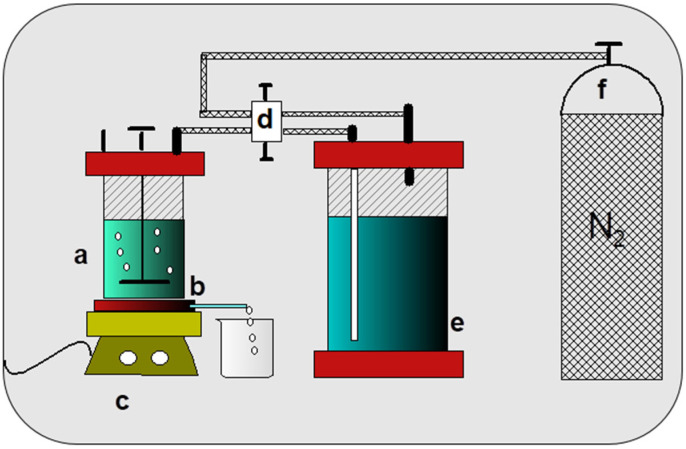
Experimental set-up of ultrafiltration system: (a) filtration cell, (b) filtrate, (c) magnetic stirrer, (d) selector, (e) reservoir, and (f) pressure source.

The UV–VIS spectra were obtained using a spectrophotometer at 25 °C between 250–700 nm using a Perkin Elmer Lambda 35, Waltham, MA, USA. The “Lyph-lock” freeze-dry system (Lab condo 6L) was used. The pH was determined with a pH-meter Hanna 211, Woonnsocket, USA. The copolymers’ thermal analysis was performed by recording TGA using a Star System 1 thermogravimetric analyzer (TGA), Barcelona, Spain, under gaseous nitrogen (at 150 and 50 cm^3^ min^−1^, respectively). The ultrafiltration system comprised a filtration cell equipped with a magnetic stirrer (Amicon 8010, 10 mL capacity), a membrane (cellulose) with a molecular weight cut-off of 10,000 Daltons (Ultracel PLCC, 5 mm diameter), a reservoir system, a flow selector, and a pressure source. Figure 11 shows the experimental set-up of the membrane filtration system used for the release studies in aqueous solution at different pH.

### 4.3. Experimental Procedures

#### 4.3.1. Preparation of the Copolymer Hydrogels

P(HEMA-*alt*-MAn) and P(HPMA-*alt*-MAn) were synthesized by free radical copolymerization (1:1 feed monomer ratio) in solution using 0.5 mol% of BPO in tetrahydrofuran (THF). The polymerization reaction was kept at 70 °C for 6 h (see Table 1).

The polymers were precipitated with n-heptane and dried under reduced pressure at 50 °C, with yields of 51.7% and 34.2%, respectively. The copolymers are soluble in dimethyl sulfoxide and exhibit swelling properties in water. Figure 1 shows the structure of the copolymers.

#### 4.3.2. Graft Reaction of the Copolymers with the Herbicide 2,4-D

The grafting procedure was carried out by obtaining 2,4-dichlorophenoxyacetyl chloride, previously carried out with SOCl_2_ to obtain the controlled-release system, purified by precipitation using hexane as solvent. According to the reaction shown in Figure 4, P(HEMA-*alt*-MAn), P (HPMA-*alt*-MAn) and 2,4-dichlorophenoxy acetyl chloride were dissolved in 5 mL of DMF as shown in Table 2. The solution was placed in a three-necked flask, which was equipped with a nitrogen inlet and outlet, a dropping funnel, a magnetic stirrer, and a thermometer. Pyridine (1 mL) was added drop-wise while stirring at approximately 0 °C. The reaction mixture was then heated to 25 °C for 5 h. After this period, the solution was poured into a large volume of cold 0.5 M HCl to precipitate the product. The product was subsequently filtered and washed several times with cold distilled water. Purification was achieved by precipitation, using THF as a solvent and cold distilled water as the precipitating agent. Finally, the product was dried under reduced pressure at 30 °C until a constant weight was obtained.

#### 4.3.3. Swelling Studies Procedure

The studies were carried out at 25 °C in buffered solutions. Before swelling and release studies, the polymer hydrogels were dispersed and purified by a membrane ultrafiltration system using a membrane with an exclusion limit of a molecular weight of 10,000 g mol^−1^. The polymeric fraction over 10,000 g mol^−1^ was freeze-dried and selected for further experiments. Dried samples of the copolymers were placed in water at a defined pH (3, 7, or 10) in buffered solutions using either 0.01 M citrate buffer (pH < 7) or phosphate buffer (pH ≥ 7). Every half hour, the hydrogel samples were quickly removed from the solution and weighed. The swelling values (*S_w_*) were calculated using the following Equation (10) [9].

(10) $$S_{W}(\%)=\frac{\left(W_{S}-W_{d}\right)}{W_{d}}\times 100$$

*W_s_* is the weight of the swollen hydrogel at an equilibrium state, and *W_d_* is the weight of the dried hydrogel (Xerogel).

#### 4.3.4. Heterogeneous Hydrolysis Procedure

This study was conducted over a month at ambient temperature. A filtration fraction was collected daily for 30 days (Z ranging from 1 to 30), using the ultrafiltration system in buffer solutions with pH levels of 3, 7, and 10. The concentration of the bioactive agent present in each filtration fraction was quantitatively assessed through UV spectroscopy, utilizing a wavelength specifically suited for detecting 2,4-D acid, measured at 282 nm. This analytical technique allowed for the precise determination of the agent’s concentration, across the different filtration fractions.

## Figures and Tables

**Table 1:** Experimental conditions for the copolymerization reaction in tethahydrofuran (THF) (5.0 mL).

Systems	HEMA		HPMA		MAn	BPO	Time	Yield
P(HEMA-*alt*-MAn)	3.40 mL	28.0 mmol			2.78 g (28.00 mmol)	69.1 mg (0.5059 mol%)	6.0 h	51.7%
P(HPMA-*alt*-MAn)			3.95 mL	28.00 mmol	2.78 g (28.00 mmol)	67.90 mg (0.4971 mol%)	6.0 h	34.2%

**Table 2:** Experimental conditions for the esterification reaction of poly(HEMA-*alt*-MAn) and poly(HPMA-*alt*-MAn).

System	Copolymer	2,4-D Chloride	DMF	Pyridine	Yield
P(HEMA-*alt*-MAn)-2,4-D	356 mg 0.021 mmol	609 mg 2.4 mmol	6.0 mL	1.0 mL	355 mg (49.7%)
P(HPMA-*alt*-MAn)-2,4-D	300 mg 0.021 mmol	547 mg 2.23 mmol	11.0 mL	1.0 mL	315 mg (55.0%)

**Table 3:** Kinetic constants (*k*_1_; in day^−1^) during the first 6 days.

System	*k*_1_ (pH 3)	R2	*k*_1_ (pH 7)	R2	*k*_1_ (pH 10)	R2
P(HEMA-*alt*-MAn)-2,4-D	0.0353	0.9802	0.1276	0.8694	0.267	0.9114
P(HPMA-*alt*-MAn)-2,4-D	0.0999	0.9673	0.1929	0.8447	0.3349	0.9176

*k*_1_ first-order release constant; *n*: order of kinetic release.

## Data Availability

The original contributions presented in this study are included in the article. Further inquiries can be directed to the corresponding author.
